# Salivary and serum periostin in kidney transplant recipients

**DOI:** 10.1371/journal.pone.0285256

**Published:** 2023-05-02

**Authors:** Seyed Mohammad Kazem Aghamir, Mahrokh Amiri, Ghodratollah Panahi, Fatemeh Khatami, Sanaz Dehghani, Mahdieh-Sadat Moosavi

**Affiliations:** 1 Urology Research Center, Tehran University of Medical Sciences, Tehran, Iran; 2 Department of Oral and Maxillofacial Medicine, School of Dentistry, Tehran University of Medical Sciences, Tehran, Iran; 3 Department of Biochemistry, School of Medicine, Tehran University of Medical Sciences, Tehran, Iran; 4 Organ Procurement Unit, Sina Hospital, Tehran University of Medical Sciences, Tehran, Iran; Health Sciences University, Faculty of Medicine, Bursa City Health Practice and Research Center, TURKEY

## Abstract

**Introduction:**

End-stage renal disease (ESRD) treatment includes dialysis and kidney transplantation. Transplant rejection is a major barrier to transplant success. One of the markers mentioned in previous studies on renal function in patients with renal failure for various reasons is periostin (POSTN). The expression of POSTN correlates with interstitial fibrosis and reduced renal function. One of the limitations in this regard is the effect of oral lesions on the POSTN level. This study was conducted aimed to measure the relationship between salivary and serum POSTN and renal function in patients with a history of a kidney transplant, taking into account all the conditions affecting POSTN.

**Methods:**

In this study, serum and saliva samples were taken from 23 transplant patients with normal function (NF) and 29 transplant patients with graft failure (GF). At least one year had passed since the transplant. Before sampling, a complete oral examination was performed. Salivary and serum POSTN was examined by ELISA. The results were analyzed by SPSS software.

**Results:**

The POSTN level in the serum of the NF group (191.00 ± 33.42) was higher than GF patients (178.71 ± 25.68), but the difference was not significant (P = 0.30). Salivary POSTN in NF patients (2.76 ± 0.35) was significantly higher than GF patients (2.44 ± 0.60) (P = 0.01).

**Conclusions:**

The superiority of saliva as a diagnostic fluid includes ease of collection and storage, and non-invasiveness, all of which can lead to the replacement of blood with this bio-fluid. The significant results of salivary POSTN may be due to the lack of serum disturbing factors. Saliva is an ultra-filtered fluid from serum and therefore there are fewer proteins and polysaccharides attached to biomarkers in saliva and the accuracy of measuring these biomarkers in the saliva is more valuable than serum.

## 1. Introduction

Chronic Kidney Rejection (CKR) is a graft failure after one year of transplant in the absence of acute rejection, drug poisoning, and other causes of nephropathy and the main cause is renal allograft failure after transplant [1]. Early diagnosis of CKR is important for the success of treatment and in fact, the focus of management of these patients is on early diagnosis, prevention, and monitoring after transplant. The high serum creatinine (Cr) and proteinuria lead to suspected CKR and require a kidney biopsy to confirm. An increase in serum Cr is not specific because may indicate various internal processes such as rejection, allograft infection, and/or transient processes such as hemodynamic effects of calcineurin inhibitors or pre-renal volume depletion. Also, Cr may not alter despite significant kidney injury [2]. Because high serum Cr and proteinuria require a kidney biopsy, and allograft biopsy has disadvantages such as the need for hospitalization, high cost, difficulty, high invasiveness, risk, sampling error, the difference in histological interpretation, and the inability to repeat, researchers have always sought solutions to monitor these patients.

A biomarker is defined as "an index that objectively measures and assesses natural biological processes, and pathogenic or drug responses to a therapeutic intervention" [3]. Also, in kidney transplant (KT), the ideal biomarker should be able to quickly, accurately, cheaply, and non-invasively identify allograft lesions and distinguish the type of injury, which has increased in recent decades and several promising blood or urinary biomarkers have been proposed.

Periostin (POSTN) is a protein in the cell-matrix with a molecular weight of 90 kDa that is expressed by various tissues during embryonic development, but in adults, it is often expressed in collagen-rich connective tissue that is subject to mechanical stress (such as periodontal ligament, periosteum, skin and heart valves). Cellular matrix proteins are a family including thrombospondins, osteopontin, SPARC, tenascin-C, connective tissue growth factors, and transforming growth factor-β (TGFβ) -inducible factor (βig-H3), of which POSTN is the newest member. POSTN expression is greatly increased following tissue damage. POSTN is directly related to the components of the extracellular matrix and it is assumed that POSTN leads to matrix integrity and affects the biomechanical properties of connective tissue [4, 5].

Over the past few years, POSTN has been recognized as a key factor in the progression of kidney diseases. This protein is secreted very briefly only during kidney development (overnight kidney) and is not normally identified in the adult kidney, but is expressed de novo in the damaged kidney (various forms of CKD). Its expression is correlated with interstitial fibrosis and reduced renal function [4, 5].

In previous studies, tissue and urine POSTN had been suggested as a predictor of graft status [1, 6]. However, histological examination is still limited due to the above reasons, and examination of urinary POSTN due to the high weight of POSTN is limited to the identification of renal dysfunction at the advanced stages of the disease and with degradation of kidney structure. Hence, further studies are needed to introduce a more valuable bio-sample in this field.

As serum can indicate systemic disorders, saliva has this ability [7, 8]. Since the whole saliva contains gingival crevicular fluid GCF, immune cells, and tissue metabolites, examination of saliva compositions can provide valuable data about biochemical markers [8]. Also, POSTN is one of the markers that can be traced in saliva. One of the advantages of salivary POSTN for use as a diagnostic factor is its easy and fast collection and positive correlation with many serum factors.

Since the effects of some oral diseases such as gingivitis and periodontitis, vesicular autoimmune diseases such as pemphigus Vulgaris or bullous pemphigoid [9], oral lichen planus [10], leukoplakia, and oral squamous cell carcinoma [11], increased gingival volume caused by drugs and sub-mucous fibrosis [12], etc. on the level of serum and salivary POSTN had been shown in previous studies, in this study for the first time we have examined salivary and serum POSTN in patients receiving KT in terms of conditions and diseases affecting POSTN including oral lesions.

In this cross-sectional study, we assessed salivary and serum POSTN of KT patients in two subgroups of NF (normal function); transplant patients with normal renal function and GF (graft failure); transplant patients with renal dysfunction, taking into account all known factors affecting POSTN, including oral and maxillofacial lesions and diseases.

## 2. Subjects and methods

This cross-sectional study conducted at Urology Research Center, Sina Hospital, Tehran University of Medical Sciences. All experimental protocols were approved by a Tehran University of Medical Sciences Ethics Committee (with the code of Ethics of IR.TUMS.DENTISTRY.REC. 149.1398 and all methods were carried out in accordance with relevant guidelines and regulations). Written informed consent was also obtained from the patients before entering the study. The study population was selected from renal transplant patients with normal function (NF) (control group), and kidney transplant patients with graft failure (GF) (case group).

Inclusion criteria: History of kidney transplantation in more than one year.

Exclusion criteria: History of any tumor or neoplasm, congenital cardiovascular disorders, pulmonary fibrosis disorders, scleroderma, allergic disorder, asthma, polycystic kidney disease, type 1 diabetes, pregnancy, autoimmune diseases, active urinary tract infection, liver diseases, other acute or chronic infections and any recent surgery.

The mouth of all patients was examined for the presence of various diseases such as gingivitis, periodontitis, and any oral lesions including oral and neck cancer, leukoplakia, lichen planus, and drug-induced gingival enlargement, and for any of these cases, the patient was excluded from this study.

To rule out gingivitis, the patient should have gingival index = 0, probing pocket depth and clinical attachment level ≤ 3 mm, no sign of bone loss, and gums have a non-inflammatory texture and natural color. 6 points around each tooth were examined (mesiobuccal, distobuccal, mid buccal, mesiolingual, distolingual, and mid lingual) [13, 14].

The examination was performed by a single examiner with a dental mirror and a Williams sterile probe, gas, and tongue blade. If a more accurate diagnosis is needed, the patient was referred for biopsy, radiographic examinations, and/or other necessary measures for the next stages of diagnosis and treatment of oral lesions (these patients were not entered our study). For this group of patients, a referral and introduction letter were written by the resident for the specialized department of the faculty. After obtaining informed consent, unstimulated saliva and blood samples were taken from all subjects as follows.

In this study, 83 kidney transplant patients were examined on an outpatient or inpatient basis. 76 patients had the inclusion criteria included in the questionnaire. Finally, 52 patients after oral examination have entered the study and analysis: 23 kidney transplant patients (44.2%) with normal function (NF) and 29 kidney transplant patients (55%) with graft failure (GF). Based on the renal function (serum creatinine measurement and GFR determination) recorded in the patient’s file at the last visit and in case of dialysis creatinine level before dialysis, the patients were divided into two groups NF (GFR above 60 and creatinine below 120 μmol) (23 patients) and GF (GFR below 30 and creatinine above 150 μmol per liter) (29 patients) [15].

### 2.1 Method of calculating the sample size

To calculate the sample size regarding EL-Dawla NMQ article [16] and considering POSTN 29.0 ± 0.02 in normal renal function and 48.0 ± 7.1 in macro-albuminuria group, error 1% and 99% power, the minimum sample size of the present study was obtained in two groups of n = 7. Given that these data do not belong to patients with current conditions, and according to the opinion of the urologists, samples have been taken up to n = 30 in each group. The calculation was done using G*Power software.

Due to the lack of effect of dialysis on POSTN, those with renal dysfunction may be on dialysis treatment. On the one hand, the serum Cr was used to divide patients into NF and GF groups (Cr in patients’ records before starting dialysis treatment). For the preparation of saliva and blood samples, to prevent changes in circadian rhythm on saliva, all saliva samples were taken in a quiet place at the same time. The unstimulated saliva sample was taken in plastic vials by spitting method after the patient avoided eating, drinking, brushing, taking medication for 90 minutes, and resting for 5 minutes. First, the patient swallowed the saliva in the mouth, and by bending the head forward and eyes open, the slightest movement of the body and head poured the saliva collected in the mouth into a calibrated vial. Saliva volume and time spent salivation were recorded to determine saliva flow and POSTN file in saliva. 5 ml of blood was taken from all patients by the clinic nurse. The samples were centrifuged at 2000 rpm for 10 min. The surface liquid of the samples was immediately frozen and stored at -70° C.

POSTN concentration of the samples was examined by ELISA ZellBio GmBH kit (Germany) (quantitative human salivary and serum POSTN test) and all stages were performed according to the manufacturer’s instructions. This kit is based on Biotin double antibody sandwich technology.

### 2.2 Statistical method

The statistical method of POSTN, which is a quantitative variable, was reported as mean and standard deviation. If the data distribution was not normal, the median and IQR were used, and for qualitative (classified) variables, frequency and percentage were used. For the mean comparison of this protein in the two groups, provided that the data are normal, the Kolmogorov-Smirnov test was used. All analyzes with 5% error were performed by SPSS Inc. software. Released 2009. PASW Statistics for Windows, Version 18.0. Chicago: SPSS Inc.

## 3. Results

In this study, 83 kidney transplant patients were examined. 76 patients met the inclusion criteria, which included the cases mentioned in the questionnaire. Finally, 52 patients were included in the study and analyzed after the oral examination. The general information of patients is given in Table 1. The age range of patients was 26–65 years and the mean age was 43.13 years. On average, 4.9 years had passed since the transplantation of patients (Table 2).

**Table 1:** Basic characteristics of subjects.

	Age±SD	Sex(F/M)	Years From Transplantation±SD	eGFR±SD	Cr±SD
NF	39.74±10.25	8/15	2.83±2.56	58.74±11.84	1.33±0.20
GF	45.83±12.67	10/19	6.67±6.0	20.78±13.63	4.51±2.71

NF: patients with Normal Function of Graft

GF: patients with Graft Failure

SD: Std. Deviation

eGFR: Estimated Glomerular Filtration Rate

Cr: Serum Creatinine

**Table 2:** Mean, median, minimum and maximum years passed from kidney transplantation in subjects.

**Years from kidney transplantation**	Mean	4.9
	Median	3
	Min	1
	Max	22

The patients underwent a complete oral examination and their saliva and serum samples were prepared, but one of the GF and NF patients refused to give a blood sample and one of the GF and NF patients refused to give a saliva sample.

**Fig 1:**
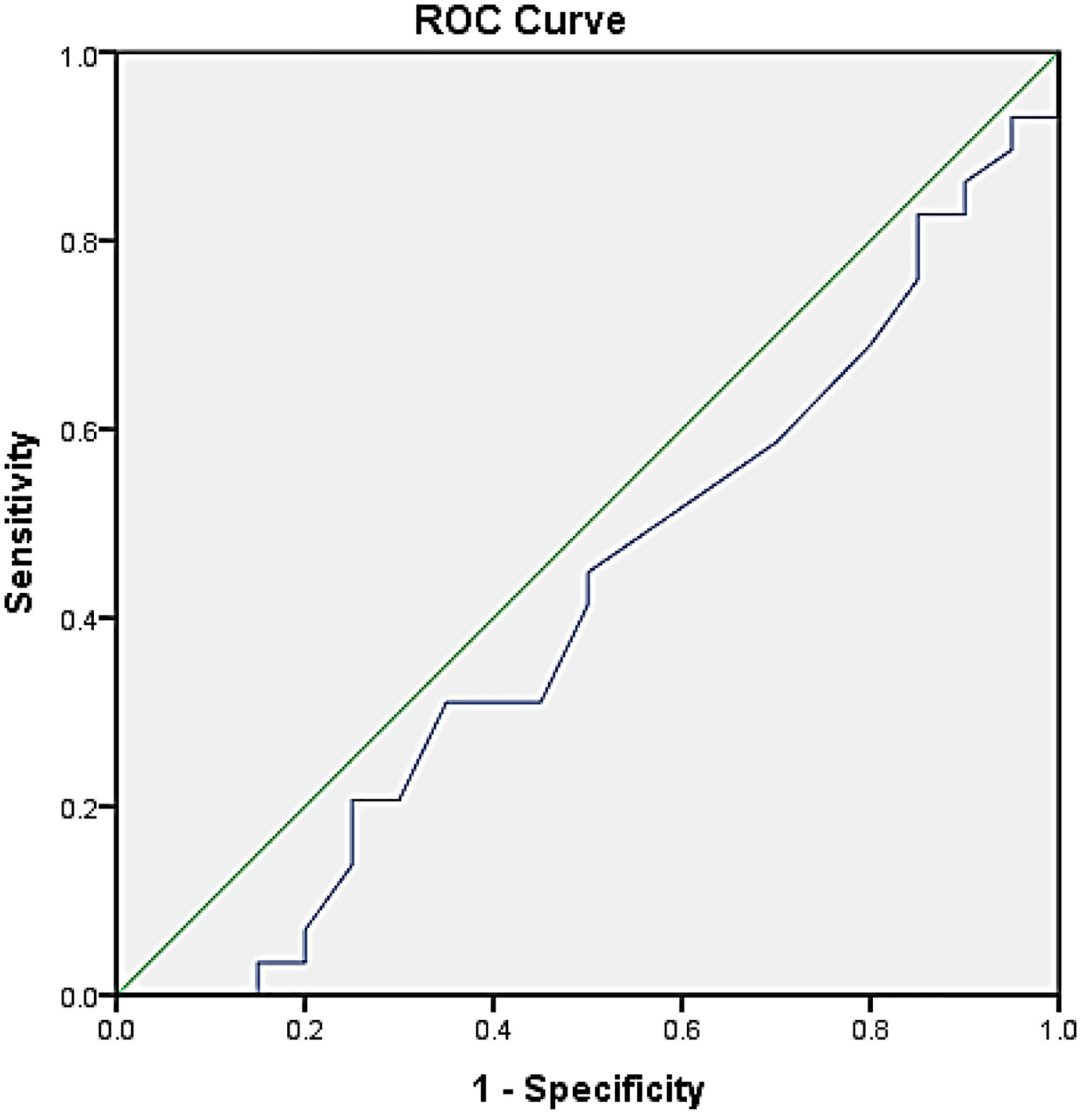
ROC test of serum POSTN.

The mean serum POSTN in NF patients was 191.00 ± 33.42 and 178.71 ± 25.68 for GF patients. The distribution of these data based on Kolmogorov-Smirnov was normal. The data were compared by t-test. In NF patients, POSTN was higher than serum POSTN in GF patients, but no significant difference was found (P = 0.30). Based on the ROC test, serum POSTN diagnostic value was measured in these patients, which was not high. Based on the ROC diagram, it can be interpreted that lower serum POSTN can be a diagnostic index of graft failure. Also, the serum cut-off point of 224.75 showed that the specificity of serum POSTN in NF patients was very low (20%) but its sensitivity in GF patients was 96.6% (high false positive) (Fig 1).

**Fig 2:**
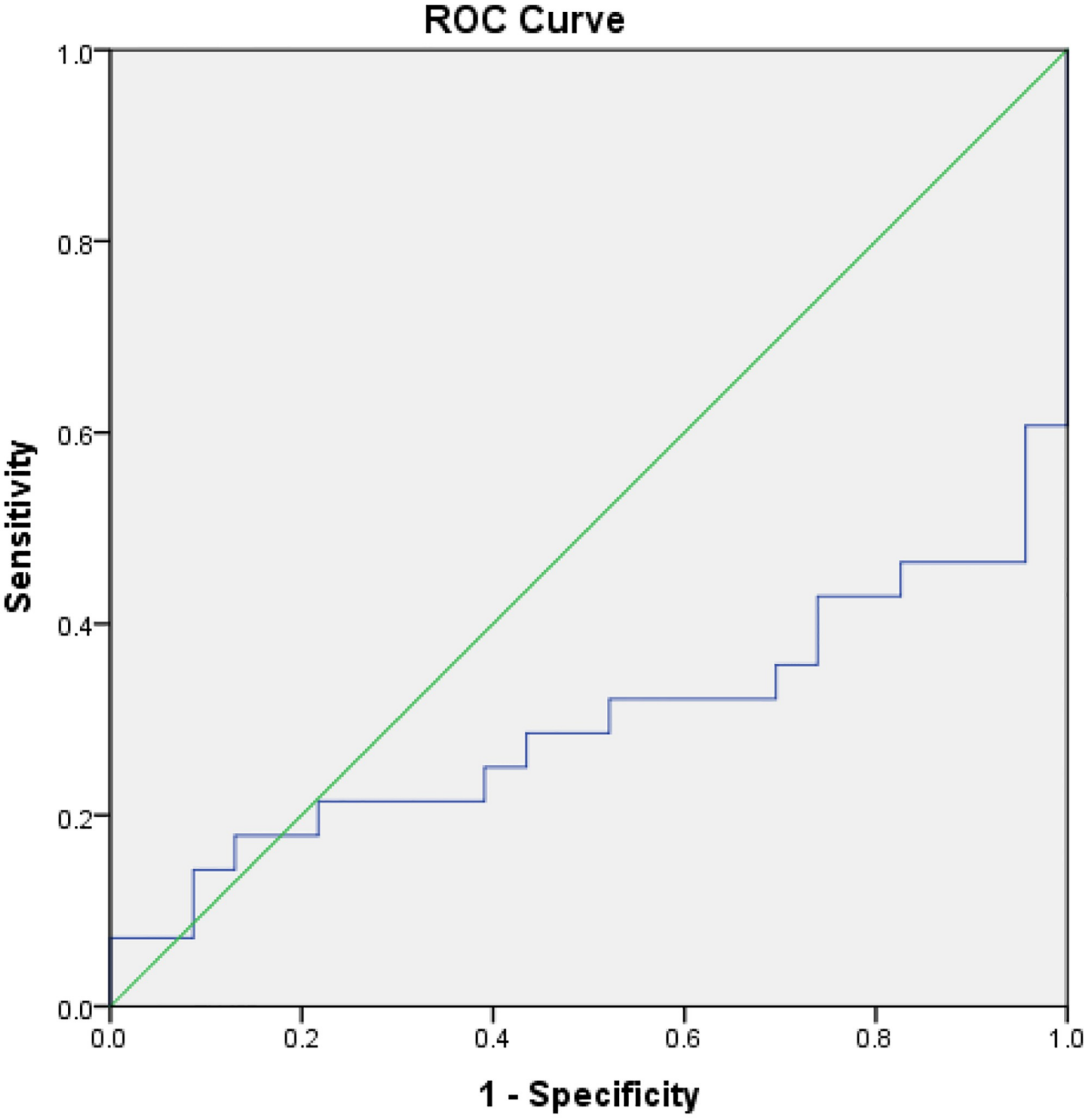
ROC test of salivary POSTN.

Salivary POSTN in NF patients was 2.76 ± 0.35 and 2.44 ± 0.60 in GF patients. After the logarithm test by Kolmogorov-Smirnov, the normal distribution of the results was measured. Therefore, a t-test was selected with a significant difference (P = 0.01). Based on the ROC test, the diagnostic value of salivary POSTN was measured in these patients, and the area under the curve was about 70% and significant (P = 0.01), indicating the high diagnostic value of salivary POSTN. According to the cut-off point of 2.34, in NF patients, salivary POSTN was 95.7% specific (low false positive), which can be a good test to confirm the diagnosis of transplant patients with normal function. Salivary POSTN also correctly diagnosed 53.6% of GF patients (low sensitivity) with a false negative in 46.4% of GF patients. It was good for confirming the diagnosis of graft failure (Fig 2).

No linear relationship was found between salivary and serum POSTN. A 0.30-correlation was found between grafts and salivary POSTN in NF patients, but it was not significant (P = 0.89).

A 0.37-correlation was found between the years after transplantation and serum POSTN in NF patients, but it was not significant (P = 0.09). A 0.10-correlation was found between the years after transplantation and salivary POSTN in GF patients, but it was not significant (P = 0.62). A 0.26 correlation was found between the years of transplantation and serum POSTN in GF patients, but it was not significant (P = 0.25).

The findings of excluded patients’ oral examinations are shown in Table 3. Also, 19.2% of patients suffered from toothache, all of whom were GF patients, which was a significant difference between included NF and GF patients.

**Table 3:** Oral findings in 24 excluded kidney transplantation patients.

Oral Disease	Percent (number of patients)
Gingivitis	29.1% (7)
Periodontitis	12.5% (3)
Gingival Hyperplasia	8.3% (2)
Leukoplakia	8.3% (2)

## 4. Discussion

In this study, it was shown that salivary POSTN in patients with a history of KT could be a marker for the prognosis of renal function. According to the World Health Organization, a biomarker is "any substance, structure, or process that can be measured in the body or its products and affects or predicts the outcome or disease" [17]. The biomarkers are divided into anatomical, serological, or physiological biomarkers [18]. In the present study, POSTN was not a valuable marker for determining renal function in patients. This can be due to many factors involved in the serum level of biomarkers. For example, previous studies have shown that factors such as a patient’s BMI or medications affect serum POSTN, but do not affect salivary POSTN [19]. Also, saliva is an ultra-filtered liquid from serum and therefore fewer proteins and polysaccharides are attached to biomarkers in saliva, and the accuracy of measuring these biomarkers in the saliva is more valuable than serum.

Since KT increases the duration and quality of life of patients, it is the best treatment option for ESRD patients. Despite the advance of KT and immunosuppressive strategies in recent decades, chronic allograft dysfunction remains the biggest concern of these patients, their families, and physicians [20]. Early diagnosis of CKR is important for the success of treatment and in fact, the focus of management of these patients is on early diagnosis, prevention, and monitoring after KT. It should be noted that neither high serum Cr nor proteinuria is adequately sensitive and specific to indicate renal impairment [21]. GFR is the most common prognostic biomarker in ESRD in clinical trials or studies. But it still does not show small changes in kidney function [21, 22]. However, currently, the monitoring of patients after KT is mostly based on blood and urine tests [7]. Kidney biopsy is controversial in terms of eligibility, cost, convenience, invasiveness, risk, sampling errors due to focal rejection features, and inability to repeat in interpretation, and can be up to 30% unclear. Also, about 80% of these methods show natural histology. Therefore, there is an undeniable need for biomarkers to quickly, accurately, cheaply, and non-invasively identify patients at risk for allograft injury. The ideal biomarker is expected to have sensitivity, specificity, positive and negative predictive value, and receiver-operating characteristic curves [23]. In the present study, according to POSTN analysis, saliva had acceptable sensitivity and specificity. Salivary POSTN is 95.7% specific (low false positive) which can be a good test for monitoring transplant patients with normal function.

The idea of using saliva biomarkers in KT patients has been mentioned in previous studies. For example, Indoxy Sulphate (IS) (a type of uremic toxin) in saliva was used to predict rejection in KT patients. In this study, as in our results, biomarkers were significant in saliva and insignificant in serum [24]. Also, a strong correlation was found between the levels of toxins (p_Cresol sulfate and IS) in serum and saliva, and in this study, a significant correlation was reported between the toxic levels of saliva and GFR [25].

Compared to the previous study, in the current study, the oral examination by an oral medicine specialist was done before entering patients to the study for excluding confounder factors related to the salivary POSTN.

An increase in POSTN expression in the glomerulus and tubular epithelium has been observed in diabetic renal pathology and is used as a marker of renal tubular injury. It is associated with worsening renal outcomes such as serum Cr, blood urea nitrogen (BUN) and eGFR, and various progressive renal dysfunction [21].

In previous studies [1], urinary POSTN had been examined in KT patients. Due to the high molecular weight of POSTN, its expression in urine indicates degradation of kidney structure and an advanced stage of transplant rejection. So, salivary and serum POSTN can be more valuable biomarkers.

Along with the increase in POSTN in the serum of NF patients, the amount of this biomarker also increased in the saliva of these patients, which was not significant in the serum but was significant in saliva. Also, a negative correlation was found between previous years of transplant surgery and salivary and serum POSTN which was not significant.

Saliva is a hypotonic solution secreted by salivary glands. The salivary glands are highly permeable, surrounded by numerous blood capillaries, and exchange many molecules, so blood flow biomarkers can be secreted into the saliva [26]. Saliva secretion and composition depend on the secretory gland, age and gender of the patient, and type of stimulus [27]. To eliminate these effects, age and gender were matched in the two groups and the whole saliva was used in both groups. Its ease of collection, painlessness, ease of storage, and non-invasiveness, all of which can lead to the replacement of blood with this bio-fluid [26]. In KT patients, collecting saliva or urine at home is more possible than taking blood [24, 27]. Saliva can facilitate biochemical and toxicological diagnosis in children and adults [27]. For example, in the follow-up of children with vesicoureteral reflux (VUR), this biomarker can eliminate the need for repetitive graphs to some extent. VUR is a disorder that causes fibrosis in the kidneys and surgery is performed at the severe stages shown on the radiographic [28].

Therefore, saliva markers will have a high potential for use in routine testing (such as salivary cortisol).

Because all the confounding factors in the POSTN level have been excluded from this study, the changes observed in this study probably indicate changes in the patient’s kidney.

The study focused on patients who passed more than one year of KT surgery. The reason for this criterion is different reasons for transplant rejection during the first year and their long-term survival such as surgical complications, recurrence of primary disease, and immunosuppression. First-year KT patients are very heterogeneous due to their instability. In some studies, this inclusion criterion has not been considered [29].

Also, it should be noted that the weight of POSTN is 90 kDa and will not be removed during dialysis and is, therefore, can measure in these patients.

Another study consistent with the present study is a study by Hachim et al. (2020) by bioinformatics analysis. The salivary and serum POSTN of asthmatic patients was examined. In this study, plasma POSTN was upregulated in patients with severe and non-severe asthma. Saliva POSTN was significantly higher in patients with non-severe asthma than in healthy ones and severe asthma patients and these patients were distinguished from each other. The salivary POSTN was also more sensitive than serum POSTN in differentiating severe from non-severe asthma. In this study, as in our study, salivary bio-fluid was the preferred biomarker over serum [19].

ELISA kit used in this study (ZellBio GmBH, Germany) is for research purposes only and not for diagnostic procedures. The serum, tissue, or salivary POSTN had not been reported in healthy people.

According to previous studies, overproduction of TGF_ẞ is one of the stimuli of POSTN secretion from the kidney. Therefore, in dialysis patients, due to the suppression of the inflammatory process caused by failure (secretion of mediators such as TGF_ẞ), the production of POSTN in the kidney is inhibited. According to previous studies, overexpression of POSTN leads to cell proliferation and increased renal fibrosis, which finally leads to reduced renal function. Therefore, it is suggested to re-examine those with very high levels of POSTN [4].

Considering the prevalence of lesions and oral diseases in kidney transplant patients and the significant prevalence of toothache in the group of patients with graft failure, we can mention the importance of the presence of dentists and oral medicine specialists along with other physicians. Based on previous studies and its confirmation in the present study, various oral lesions occur in post-transplant patients, a significant percentage of which can be treated by timely examination and identification. In the center where the present study was conducted, dental and oral health examination and monitoring are performed only before the transplant operation and there is no such monitoring during the post-transplant period. Therefore, this amount of exclusion of the patient does not necessarily happen in other medical centers and patients, especially if the patients are undergoing dental examination. Also it is suggested for future studies investigate the relationship between drug level and oral tissue changes in transplant patients.

In addition to considering chronic renal rejection (more than one year), it is suggested to conduct a study to examine POSTN in a group with the rejection of less than one year. In future studies, it is better to examine tissue POSTN along with salivary and serum POSTN. It is also suggested to consider a group of healthy people along with transplant patients.

## 5. Conclusion

Salivary POSTN in patients with a history of KT could be a marker for the prognosis of renal function. The superiority of saliva as a diagnostic fluid may lead to the replacement of blood with this bio-fluid. The significant results of salivary POSTN may be due to the lack of serum disturbing factors. Saliva is an ultra-filtered fluid from serum and therefore there are fewer proteins and polysaccharides attached to biomarkers in saliva and the accuracy of measuring these biomarkers in the saliva is more valuable than serum.
